# ^31^P and ^1^H Nuclear Magnetic Resonance Spectroscopy of Blood Plasma in Female Patients with Preeclampsia

**Published:** 2012-12

**Authors:** Susanne Schott, Josef Hahn, Christian Kurbacher, Detlef Moka

**Affiliations:** 1 *Department of Nuclear Medicine, University of Cologne, Cologne, Germany;*; 2 *Institute of Inorganic Chemistry, University of Cologne, Cologne, Germany;*; 3 *Department of Obstetrics and Gynecology, University of Cologne, Cologne, Germany;*; 4 *Clinic for Nuclear Medicine, University Hospital Essen, Essen, Germany*

**Keywords:** preeclampsia, gestosis, lipid metabolism, ^31^P and ^1^H-magnetic resonance spectroscopy

## Abstract

**Objective:**

Using ^31^P and ^1^H magnetic resonance spectroscopy to measure phosphorus- and hydrogen-containing metabolites, this study aimes to investigate whether or not women with preeclampsia have detectable systemic abnormalities concerning certain components of the blood plasma.

**Methods:**

Plasma was obtained from two groups of women: Group 1 with preeclampsia (n=10) and Group 2, as a control group with no complications during pregnancy (n=10). Plasma analysis were performed using *in-vitro* ^31^P and ^1^H nuclear magnetic resonance spectroscopy.

**Results:**

^31^P nuclear magnetic resonance (NMR) spectra showed significantly higher levels of lysophosphatidylcholine 1 in the plasma of the patients in Group 1, along with significantly decreased levels of lysophosphatidylcholine 2 and phosphatidylinositol. However, the total amount of phospholipids did not differ significantly between the groups. In addition, the ^1^H NMR spectra showed a significantly lower level of HDL in samples from Group 1, and a trend towards higher plasma levels of VLDL 2 and LDL 2 in the same group.

**Conclusion:**

This study supports the theory that preeclampsia is a disorder in phospholipid metabolism in which malfunctioning of cellular membranes seems to play a major pathogenic role.

## INTRODUCTION

Gestosis, including pregnancy-induced hypertension, preeclampsia (PE) and eclampsia, is a multisystem disorder that affects and therefore complicates up to 10% of pregnancies worldwide (1). Hemolysis, elevated liver enzymes and low platelet count, first described as HELLP-syndrome by Weinstein (2), is a severe form of preeclampsia posing unpredictable risks to mother and fetus. Up to 19% of pregnancies affected by preeclampsia may be further complicated by HELLP-syndrome (3). In such cases, maternal mortality has been reported to be as high as 24% and perinatal mortality as high as 30-40% (4). The only cure for HELLP-syndrome is delivery, yet this is a double-edged sword at gestational ages at which neonatal outcome is poor. Numerous studies have been conducted in an attempt to improve maternal and fetal outcome and to identify the pathogenesis of this disease in order to improve diagnosis, management and treatment. Furthermore, it would be of major interest to find a substance that could be used as an early marker in the diagnosis of preeclampsia and HELLP-syndrome in order to prevent severe complications as early as possible. However, the etiology and pathogenesis of HELLP-syndrome and gestosis remain unknown. It is thought that genetic predisposition and immune maladaption contribute to alterations of both the cellular membrane and coagulation systems (5-8). There is increasing evidence that malfunction of the coagulation system leads to placental micro-embolism and infarction and thus to inadequate maternal-fetal circulation (9). Moreover, some publications have noted alterations in the composition of phospholipids as well as significant changes in the fatty acid profile in the blood of women affected by preeclampsia or HELLP-syndrome, which however remained unexplained until now (10, 11).

High resolution (HR) ^31^P and ^1^H nuclear magnetic resonance (NMR) spectroscopy provide powerful means to fill this basic knowledge gap by measuring steady-state levels and fluxes of metabolites in the metabolic pathways of lipid, phospholipid and cell membranes. The NMR methods have been used in analytical chemistry for years to identify and classify substances and are alternative non-invasive and non-destructive methods of investigating the metabolic and biochemical status and composition of biological samples. The recent rapid development of NMR spectroscopy techniques, especially of high resolution (HR) spectra, has led to improvements in the assignment of resonance and quantification of results (12). The spectra are now of high specificity and yield direct molecular information. Both sensitivity and spectral resolution depend on the field strength applied (13). HR - ^31^P and ^1^H NMR spectroscopy measure concentrations of multiple phosphorus- and hydrogen-containing metabolites and can provide valuable information on the biology and pathology of a disease and may provide fingerprints for disease classification (13).

The aim of the present study therefore was to investigate systemic alterations of gestosis/preeclampsia by quantifying and characterising phospholipid, amino acid and fatty acid profiles.

## METHODS

Patients suffering from preeclampsia and healthy pregnant women, as control group, were informed about the study and invited to participate during their admission to the Department of Obstetrics and Gynaecology at the University Hospital of Cologne. Ten patients were included into the group of women developing a preeclampsia (Group 1). The diagnosis of preeclampsia was made on the basis of hypertension (systolic pressure > 140 mmHg or diastolic pressure > 90 mmHg) and proteinuria, a manifest HELLP-Syndrome could be excluded based on laboratory findings (no hemolysis, elevation of serum glutamic pyruvic transaminase or thrombocytopenia could be detected). Ten healthy, pregnant women served as control group (Group 2).

The study was approved by the Hospital Human Rights Committee (Institutional Review Board) and written informed consent was obtained from all patients.

Data were statistically analysed by the *Student’s* t-test, and significance levels set at *p*<0.05, incorporating the Welch correction.

Immediately before delivery, peripheral venous blood (10 ml) was collected from all women in a heparinized Vacutainer and centrifuged for 10 minutes at 3000 × g. The plasma was stored at –80°C until required for the NMR-experiment.

The plasma for the ^31^P-NMR spectra was placed in a 10 mm phosphorus NMR tube with N-phosphonomethylglycine (5 mmol/l) added as a standard and sodium cholate as a detergent solution.

Plasma for the ^1^H NMR spectra was placed in a 5 mm inverse NMR tube. The standard solution for the ^1^H NMR spectroscopic measurements contained 25.3 mmol/l of tetradeuterotrimethylsilylpropionate as an internal reference compound for quantification purpose, D_2_O as a solvent and NMR lock compound in 25 ml solution.

## NMR SPECTROSCOPY

### *In vitro* ^31^P MR spectroscopy

The one-dimensional 121.49 MHz (7 Tesla) ^31^P NMR spectra of the blood plasma were recorded on a Bruker AMX 300 spectrometer, sample temperature being held at 310 K, using a 10 mm phosphorus probe head. Inverse-gated ^1^H decoupling (waltz 16) was applied to eliminate ^31^P-^1^H coupling and to avoid NOE build up. Other acquisition parameters were as follows: pulse length 3.8 μs (30°), sweep width 3906 Hz, time domain 16 k data points, number of scans 5000, repetition time 3.5 s. Longitudinal ^31^P relaxation times (T_1_) of the reference compound and the phospholipids contained in standard plasma samples under standard conditions were determined using an inversion recovery pulse sequence. The resulting T_1_ values were 3.4 s for the reference compound and 1.3–2.0 s for the phospholipids (see Table 1). Trial measurements showed that a repetition time of 3.5 s at a pulse width of 3.8 μs (30°) was sufficient for prevention of saturation effects. The spectra were processed using WIN-NMR 5.1^®^ Bruker Analytische Meßtechnik GmbH. The FID´s were Fourier transformed after zero filling to 32 k data points and application of an exponential function with a line broadening constant of 1 Hz. Chemical shift assignments were referenced relative to 85% orthophosphoric acid at 0 ppm. The peak areas were determined by iterative deconvolution using a program of the PERCH project (Kupio, Finland) (14).

**Table 1:** ^31^P nuclear magnetic resonance spectroscopy data in patients suffering from preeclampsia (Group 1) and healthy, pregnant women (Group 2). Comparisons between Groups 1 and 2 by means of Students t-test to determine significance (*p*<0.05)

Substance	Chemical Shift	Mean Concentration in μmol/L ± SD		*P*
		Gestosis	Controls	

**PI**	0.09	55.9 ± 21.2*	157.6 ± 104.0*	0.022
**LPC 2**	0.19	21.4 ± 12.0*	47.1 ± 20.2*	0.007
		**mmol/l ± sd**		

**PE + SM**	0.52	0.71 ± 0.21	0.67 ± 0.15	0.638
**LPC 1**	0.35	0.31 ± 0.03*	0.17 ± 0.07*	<0.001
**PC**	-0.09	2.41 ± 0.62	2.22 ± 0.44	0.473
**Total PL**		3.51 ± 0.82	3.26 ± 0.65	0.506

PE + SM, phosphatidylethanolamine and sphingomyelin; LPC, lysophosphatidylcholine; PI, phosphatidylinositol; PC, phosphatidylcholine.

### *In vitro* ^1^H MR spectroscopy

The one-dimensional 500 MHz (11.7 Tesla) ^1^H MR spectra of the blood plasma were recorded on a Bruker DRX 500 spectrometer, sample temperature being held at 310 K, using a 5 mm inverse probe head. The spectra were recorded using a pulse sequence that suppresses the water signal by means of presaturation (Bruker© program *noesypr1d*) which turned out to be the best method for suppression of the water signal. The SFO1 (standard frequency for measurement) was set on the water signal. Other acquisition parameters were as follows: pulse length 12.0 μs (90°), sweep width 6009 Hz, time domain 32 k data points, number of scans 256, repetition time 5.7 s. Longitudinal ^1^H relaxation times (T_1_) of the reference compound and the lipids contained in standard plasma samples under standard conditions were determined using an inversion recovery pulse sequence. The resulting T_1_ values were 0.6 s for the reference compound and 0.3-0.4 s for the signals of the lipids. The spectra were processed using X-WIN-MR 2.0^®^ Bruker Analytische Meßtechnik GmbH. Chemical shift assignments were referenced relative to the reference compound at 0 ppm. The peak areas were determined by iterative deconvolution using a program of the PERCH project (Kupio, Finland) (14).

## RESULTS

Plasma samples of ten patients with preeclampsia and ten healthy pregnant women were studied. The median maternal age was 33.4 years (range 19–38). The median week of pregnancy for developing preeclampsia was 33.2 (range 25–40). The patients in Group 1, all underwent delivery by Caesarean section.

**Figure 1:**
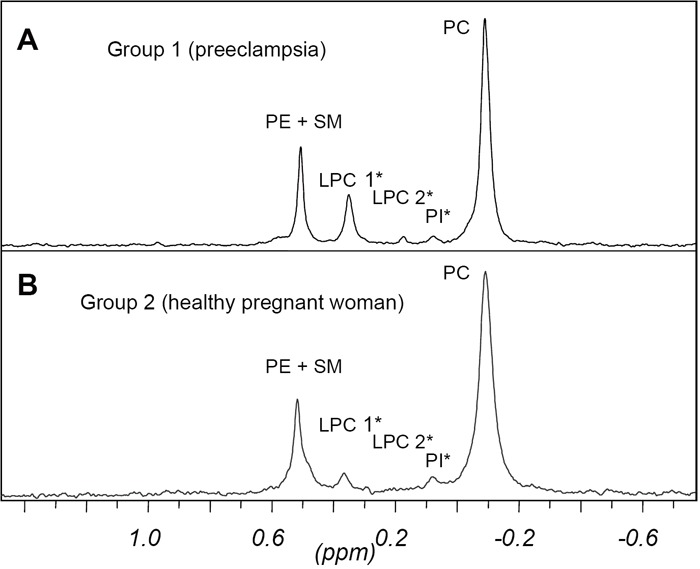
One-dimensional 31P NMR blood spectra in pregnant women. A, ^31^P NMR spectrum in a patient suffering from preeclampsia. B, ^31^P NMR spectrum in a healthy, pregnant woman (*shows significant differences in concentrations of substances). Chemical shift assignments were referenced relative to 85% orthophosphoric acid at 0 ppm.

Figure 1 shows a typical ^31^P NMR spectrum obtained from human blood plasma treated with sodium cholate. This detergent causes the formation of mixed micelles thus reducing the line-width of the signals. The partial spectra show the region of the phospholipids located upfield. The peaks for inorganic phosphate and for the internal reference compound added to quantify the phospholipid concentrations are not shown. The experiments are the typical HR- ^31^P nuclear magnetic resonance (NMR) spectra in patients suffering from preeclampsia (Group 1) and healthy pregnant patients (Group 2). Table 1 shows the chemical shifts (δ) for resonance assignment und summarizes the results of the experiments.

The resonances were assigned to the various phospholipid classes, namely phosphatidylethanolamine plus sphingomyelin (PE + SM), 1- and 2-acyl-lysophosphatidylcholine (LPC1 and LPC2, resp.), phosphatidylinositol (PI) and phosphatidylcholine (PC). The assignments were confirmed by adding standard phospholipids to the plasma samples and observing the increase in signal intensity.

As only few samples contained phosphatidylserine (PS) and cardiolipin (CL) at a concentration that could be detected by NMR, these compounds were not quantified.

Some plasma samples moreover showed peaks indicating degradation products of phospholipids (eg. glycerophosphate). Peaks found in plasma samples from gestosis patients as well as from control group however were neglected due to their low intensity. All other signals could be quantified with sufficient reproducibility by comparing the peak areas of the phospholipids with those of the reference compound that was present at a constant concentration.

Although LPC2 and PI give small signals (signal/noise ratio from 5 up to 21, through iterative deconvolution, integration reached sufficient reproducibility. The PL concentrations of the two patient groups did not differ significantly.

However, in patients with preeclampsia, significantly higher levels of LPC 1 and significantly lower levels of LPC 2 and PI were detected.

**Figure 2:**
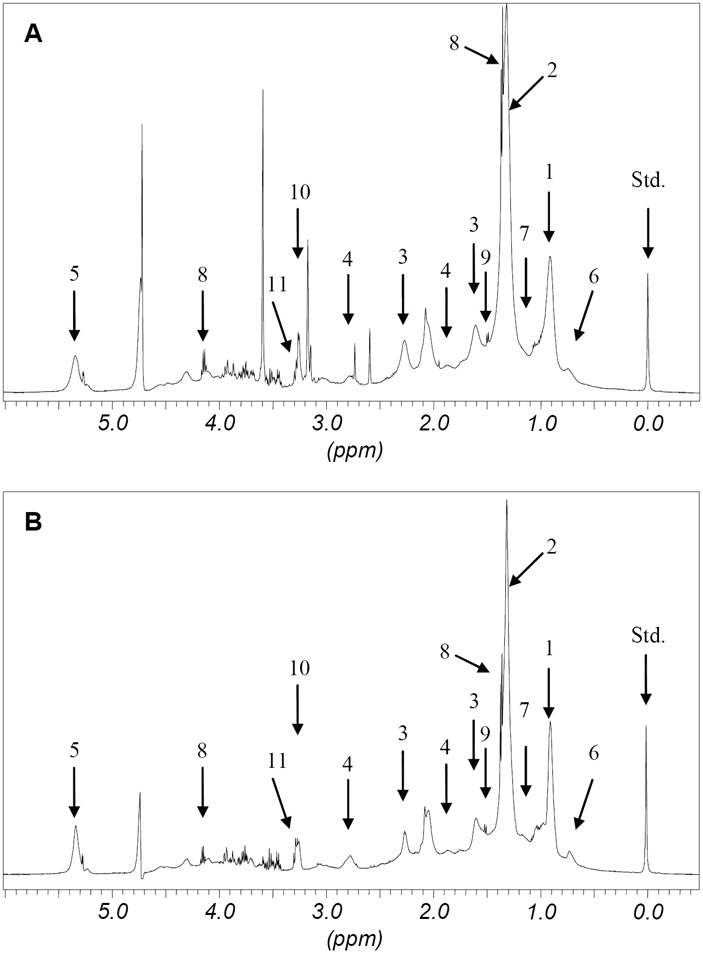
Partial ^1^H NMR spectra in a pregnant women. A, ^1^H NMR spectrum in a patient suffering from preeclampsia. B, ^1^H NMR spectrum in a healthy, pregnant woman. The assignments of the major NMR resonance are given in Table 2. (Standard solution (Std.) tetradeuterotrimethylsilylpropionate).

Figure 2 shows typical examples of spectra of HR ^1^H NMR spectroscopy. The results after assignment of the major resonance and quantification of metabolites are given in Table 2. In the spectra we could identify several resonances of metabolites featured in lipid and phospholipid metabolism. Furthermore, we identified a number of amino acid resonance based on spin-spin coupling patterns and chemical shift values. The amino acids appear to exist as isolated molecules based on chemical shifts, especially concerning the alpha carbon protons. However, the metabolite pattern did not differ significantly between patients with and without gestosis. Thus, there was no evidence of any alterations in the phospholipid and amino acid/protein pathways for women with preeclampsia. With regard to lipid structures, wide variability was found within the LDL and VLDL fractions. However, these differences were not significant. The only significant finding was a decrease in HDL concentrations in patients suffering from preeclampsia.

**Table 2:** Concentrations of different metabolites of lipid and protein pathways in gestosis patients (Group 1) and healthy, pregnant women (Group 2). Please note that only HDL concentrations were significantly lower in Group 1

	Substance	Median Concentrations in mmol/L ± Standard Deviation		p
		Preeclampsia	Controls	

1	VLDL 1 → CH_3_(CH_2_)_n_	1.09 ± 0.11	1.10 ± 0.11	0.860
	LDL 1 → CH_3_CH_2_CH_2_HC=			
2	VLDL 2 → (CH_2_)_n_	4.45 ± 1.06	3.42 ± 0.67	0.051
	LDL 2 → CH_2_CH_2_COOR			
3	CH_2_CH_2_COOR (fatty acids)	0.24 ± 0.13	0.24 ± 0.07	0.937
4	CH=CH-CH_2_-CH=CH-CH (originating from fatty acids)	0.18 ± 0.04	0.20 ± 0.03	0.341
5	Unsaturated fatty acids	1.28 ± 0.30	1.23 ± 0.19	0.696
		**μmol/l ± SD**		

6	CH_3_ group of cholesterol in HDL	37.3 ± 8.0^a^	48.5 ± 5.1^a^	0.010
7	Valine −CH_3_	33.4 ± 3.9	37.4 ± 3.0	0.060
8	Lactate −CH_3_	85.1 ± 69.5	108.1 ± 33.7	0.434
9	Alanine −CH_3_	9.5 ± 6.4	9.4 ± 2.6	0.951
10	Choline −N^+^(CH_3_)_3_	56.3 ± 7.7	63.3 ± 7.7	0.136
11	Glucose with Choline	21.8 ± 14.6	45.6 ± 23.2	0.066

## DISCUSSION

The results obtained in this study support previous work, suggesting that a dysfunction of cellular membrane systems as well as an imbalance of the fatty acid profile occur in gestosis and preeclampsia and that these dysfunctions lead to systemically detectable alterations (15). As certain pathologic processes can cause simultaneous changes in multiple metabolites, changes in single metabolites may not fully represent the underlying process. However, differences in a large number of metabolites including lipids and phospholipids were observable. We succeeded in characterising and quantifying these differences using high resolution ^31^P and ^1^H NMR spectroscopy of blood plasma. Analysis of ^31^P spectra in particular, indicated that some cellular membrane systems might be affected in this multifactorial disease. The phospholipids detected by the ^31^P experiment are all an integral part of membrane structures essential to normal functions such as exchange of metabolites and maintenance of cellular integrity. There have been several investigations into lipid status, lipid peroxidation (10) and changes in fatty acid profile, and it has frequently been proposed that lipid peroxidation in membranes and peroxidation of polyunsaturated fatty acid chains will disrupt membranes and reduce fluidity (16). Both increased and decreased phospholipids have been detected in different studies (17) but the levels of fatty acids have been found to be elevated in the majority of studies (18). However, the only significant alteration found in the present study in ^1^H NMR spectroscopy was in the amino acid and the HDL fraction. The differences observed in carbohydrate metabolism and parts of fatty acid pathways were statistically insignificant. Furthermore, we found no correlation between the severity of gestosis and the extent of alterations in membrane metabolism.

Nevertheless, a change in the degree of unsaturation of fatty acyl chains of phospholipids could be expected to alter the physical properties of, for instance, placenta cell membranes. A more fluid lipid membrane phase could then increase the likelihood of systemic alterations.

## CONCLUSION

This study supports the theory that preeclampsia is correlated with a disorder in membrane/phospholipid metabolism. Systemic metabolic profiling using high resolution NMR spectroscopy is a powerful analytical tool, offering a rich source of information on the biochemistry of gestosis. Although significant systemic alterations can be detected in case of preeclampsia, it remains unclear whether these changes are a consequence of pregnancy-induced hypertensive disease or whether they play a key role in the pathogenic process (19, 20). Further studies are needed to clarify at what stage of the disease deteriorations in phospholipid and fatty acid profiles appear.

## ABBREVIATIONS

PE: phosphatidylethanolamine
SM: sphingomyelin
LPC: lysophosphatidylcholine
PI: phosphatidylinositol
PC: phosphatidylcholine
PS: phosphatidylserine
δ: chemical shift (ppm)
HDL: high-density lipoprotein
LDL: low-density lipoprotein
VLDL: very low-density lipoprotein
HR NMR: high resolution NMR
n.s.: not significant
